# Plant Silicon and Phytolith Research and the Earth-Life Superdiscipline

**DOI:** 10.3389/fpls.2018.01281

**Published:** 2018-09-05

**Authors:** Ofir Katz

**Affiliations:** Dead Sea and Arava Science Center, Tamar Regional Council, Israel

**Keywords:** plants, silicon, phytolith, earth system, superdiscipline

## Disciplinary origins of plant silicon and phytolith research

Plant silicon and phytolith research stands as a good example for how a single phenomenon or theme is studied by scholars from multiple disciplines, and for how knowledge flows among disciplines. At its very core and origins, plant silicon and phytolith research lies in traditional botany, since it studies the occurrence and role of silicon and phytoliths within plants and among plant groups (e.g., Hodson et al., 2005; Katz, 2014, 2015; Strömberg et al., 2016) and can be potentially used to improve taxonomy and systematics by providing more characters to be included in analyses (e.g., Prychid et al., 2004; Katz, 2014, 2018a). Nevertheless, plant physiologists study the mechanisms of silicon uptake, transport and accumulation within plants (e.g., Peleg et al., 2010; Mitani-Ueno et al., 2014; Ma and Yamaji, 2015; Kumar et al., 2017), chemists study the mechanisms of its deposition (e.g., Currie and Perry, 2007; Patwardhan et al., 2012), ecophysiologists identify silicon's and phytoliths' functions within plant tissues (e.g., Fauteux et al., 2005; Liang et al., 2007; Epstein, 2009; Guntzer et al., 2012; Cooke and Leishman, 2016; Coskun et al., 2016) and ecologists study how silicon and phytoliths interact with herbivores (e.g., Massey and Hartley, 2006; Katz et al., 2014; Hartley, 2015; Frew et al., 2016) and shape plant communities (e.g., Jacobs et al., 2013; Schoelynck et al., 2014; Cooke et al., 2016), ecosystems (e.g., Cooke and Leishman, 2011; Cooke et al., 2016; Schoelynck and Struyf, 2016), and even biomes and the entire ecosphere (e.g., Carey and Fulweiler, 2012, 2016; Song et al., 2012, 2017; Katz, 2018b).

Within Earth sciences, biogeochemists study the physics and chemistry of plant silicon and phytoliths, including their dissolution (e.g., Fraysse et al., 2009; Cabanes and Shahack-Gross, 2015) and chemical and isotopic composition (e.g., Hodson et al., 2008; Kamenik et al., 2013; Alexandre et al., 2015). Others study the silicon cycle (e.g., Alexandre et al., 2011; Carey and Fulweiler, 2012, 2016; Song et al., 2012, 2017) and its connections with other biogeochemical cycles (e.g., Street-Perrott and Barker, 2008; Carey and Fulweiler, 2012, 2016; Song et al., 2012, 2017; Alexandre et al., 2015; Cornelis and Delvaux, 2016). Plant silicon and phytoliths are also often used in geoarchaeology to infer past human life (e.g., Tsartsidou et al., 2008; Lancelotti et al., 2014; Hart, 2016), as well as in paleontology to reconstruct ancient vegetation and ecosystems (e.g., Strömberg et al., 2007; Albert and Bamford, 2012) and to trace the evolution of plants and animals (e.g., Prasad et al., 2011; Strömberg, 2011; Katz, 2015; Strömberg et al., 2016).

Thus, plant silicon and phytolith research demonstrates the integration of knowledge from both Earth and life sciences. The plant silicon and phytolith research community studies the effects of plant silicon uptake on other organisms, ecosystems and biogeochemical cycles in tandem with the effects of other organisms, ecosystems and biogeochemical cycles on plant silicon uptake. Likewise, members of the community use geoarchaeological and palaeontological methods to understand the evolution and history of plants and animals, while using knowledge of the evolution of plants and animals to understand changes in the geosphere and Earth themselves. While all these themes are intimately connected, share many theoretical and methodological aspects, and constitute a single research topic, only rarely do we see a researcher or a research group that covers a considerable portion of this wide range. One possible reason for this is that the question one asks, the methods one employs to answer them, and the interpretations of these results are strongly influenced by one's parent discipline. Many of us study plant silicon and phytoliths as part or in addition to other themes within our parent disciplines, thus hindering the formation of a common meeting ground or language for plant silicon and phytolith researchers from various parent disciplines. The compilation of the International Code for Phytolith Nomenclature (Madella et al., 2005) is an advancement toward solving part of this problem, albeit somewhat limited to more technical rather than theoretical issues.

**Figure 1:**
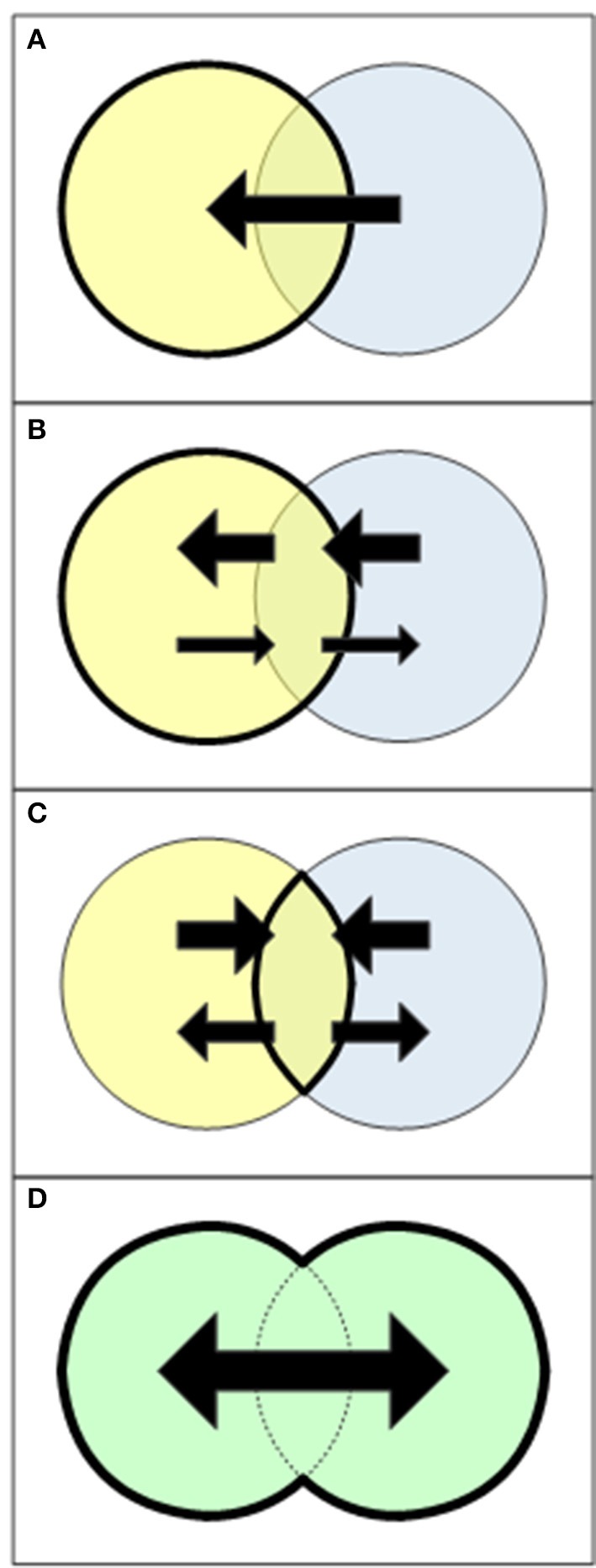
Four models for transfer and sharing among disciplines (Klink et al., 2002; Krishnan, 2009). **(A)** Cross-disciplinary knowledge transfer: Scholars from one discipline (yellow) use knowledge or methods from another discipline asymmetrically and unidirectionally. **(B)** Multidisciplinary collaboration: One discipline (yellow) initiates a research programme, on which other disciplines work independently. Synthesis is carried out almost solely by the initiating discipline, and although knowledge transfer is not unidirectional, it is asymmetrical. **(C)** Interdisciplinary framework: Several disciplines share a theoretical framework. All disciplines contribute knowledge to the shared framework and take part in synthesis. Knowledge flows symmetrically, but through a mediating intersection. **(D)** A superdiscipline: Disciplines are rearranged by relaxing boundaries among them and thus looking at the union rather than at the intersection. Each discipline bears an equal weight and knowledge flows in all directions (ideally) free of constrains.

By remaining bound to parent disciplines, we sentence our field to remain adjacent to the mainstream (rather than within it) and led by parent disciplines and their agendas. Instead, we should form a greater integrated framework that links our parent disciplines, extends their scopes, increases dialogue among them, and achieves high-order knowledge transfers among them (Figure 1). This is possible now more than ever. Since Earth and life sciences are merging, our field that sits between them can gain a rightful place at the center stage of a new emerging superdiscipline.

## Silicon and phytolith research within the interdisciplinary earth-life sciences merger

As science progresses, so do models of knowledge transfer (Krishnan, 2009). The simplest model is cross-disciplinary knowledge transfer (Figure 1A), in which knowledge from one discipline is borrowed by researchers from another discipline, without true collaboration or dialogue among disciplines. This model is very uncommon because of its inherent shortcoming: that people outside a discipline use knowledge although they have lesser understanding of its underlying assumptions and theories or of specific methodologies (Keene, 1983; Krishnan, 2009). The use of this model nowadays is limited strictly to methodological technicalities.

A second, common model is of multidisciplinary collaboration (Figure 1B), in which one discipline initiates a research programme, on which research teams from other disciplines work independently. The initiating discipline is responsible for synthesis and gains most of the knowledge, while the other disciplines gain less knowledge (often methodological knowledge only). Examples for multidisciplinary collaborations in plant silicon and phytolith research include the use of phytolith analysis to increase botanical knowledge in archaeology (e.g., Albert et al., 1999; Tsartsidou et al., 2008; Lancelotti et al., 2014; Hart, 2016) and palaeoecology (e.g., Albert and Bamford, 2012). Others revealed parts of evolutionary history through new insights into plant physiology and ecology (e.g., Strömberg et al., 2007; Prasad et al., 2011; Strömberg, 2011; Katz, 2015, 2018a). Vice versa, some scholars use plant silicon and phytoliths to identify possible external evolutionary stimuli that may provide insight into the function of plant silicon and phytoliths (e.g., Katz, 2014, 2015; Strömberg et al., 2016). Finally, phytolith chemistry contributes to our understanding of silicon dissolution in soil and transport in ecosystems (Fraysse et al., 2009; Alexandre et al., 2015; Cornelis and Delvaux, 2016).

A third, more complex model is the interdisciplinary framework (Figure 1C), in which researchers from various disciplines contribute and gain relatively equally, but all knowledge transfer is carried out through a shared theoretical framework. Two interdisciplinary frameworks that are of special interest for plant silicon and phytolith researchers are Earth System Science (ESS) and plant functional diversity. ESS is an interdisciplinary framework that attempts “to obtain a scientific understanding of the entire Earth System on a global scale by describing how its component parts and their interactions have evolved, how they function, and how they may be expected to continue to evolve on all time scales” (Earth System Science Committee, 1986) by applying methods and concepts from systems and complexity theories. ESS is therefore a merger of Earth and life sciences that uses systems and complexity theories as the common ground. Both paleontology and ecosystem ecology can be seen as subdivision of ESS, the former focusing on evidence for the evolution of the entire Earth System and the latter focusing on the direct interactions of Earth and life components within ecosystems, hence relying on emergence theory and ecosystem theory (respectively) as subsets of systems and complexity theories. Some studies of the silicon cycle and its interactions with the carbon cycle have quite explicitly used systems and complexity theories (Alexandre et al., 2011; Carey and Fulweiler, 2012, 2016; Cornelis and Delvaux, 2016), and thus represent an integration of plant silicon and phytolith research within the ESS framework.

Plant functional traits are quantitative traits whose values are affected by environmental variables and affect plant, community and ecosystem properties and functioning (Garnier et al., 2016). When discussing ecosystem functions like elemental cycling, plant functional diversity is an interdisciplinary framework that connects Earth and life sciences, with plant functional traits as the common ground that mediates the effects of Earth components on plants and the effects of plants on the ecosystem, again greatly relying on systems theory. Therefore, ESS studies and models can improve if they take into account plant functional traits and types (Beerling, 2007; Van Bodegom et al., 2012; Wullschleger et al., 2014). Although often ignored by mainstream plant functional diversity literature, plant silicon and phytolith contents are gaining increasing recognition as a plant functional trait, and are now known to be involved in plant responses to their environment and plant effects on the environment (Cooke and Leishman, 2011; Carey and Fulweiler, 2012; Song et al., 2012, 2017; Katz, 2014, 2015, 2018b; Schoelynck et al., 2014; Cooke et al., 2016; Schoelynck and Struyf, 2016). Therefore, plant silicon and phytolith research is a part of the interdisciplinary ESS framework.

## An earth-life superdiscipline–A promising future

These three aforementioned models, and especially multidisciplinary collaborations and interdisciplinary frameworks, have served scientists very well, including in merging knowledge from Earth and life sciences and in plant silicon and phytolith research. However, they are not without shortcomings, including the asymmetry of knowledge transfer, the adherence to certain framing theories, and the limited integration that stems from maintaining boundaries among disciplines.

These shortcomings are overcome in the most advanced model of knowledge transfer and sharing among disciplines, the superdiscipline (Figure 1D), in which boundaries among disciplines are relaxed and knowledge flows freely within the greater domain of the superdiscipline, unbounded to any discipline or framing theory. Although relaxing disciplinary boundaries without the mediation of framing theories is difficult, it is very promising when attempting to answer big, complex, discipline-transgressing and irreducible questions (Krishnan, 2009). The seeds for a merged Earth-life superdiscipline have been sown many years ago. Ecosystem ecology, ESS and plant functional diversity represent great advancements in this direction, yet as interdisciplinary frameworks they are bound to the intersections of the parent disciplines and to the framing of systems and complexity theories. The road to a true Earth-life superdiscipline lies, at least in part, in removing these boundaries, as Beerling (2007) has nicely demonstrated in his book *The Emerald Planet*, which introduces a synthesis of plant physiology, paleontology and atmospheric sciences.

Somewhat ironically, the fact that plant silicon and phytolith research is adjacent to the mainstream means that it is less constrained than existing interdisciplinary frameworks, and therefore freer to achieve superdiscilinarity and have a leading role in the formation of an Earth-life superdiscipline. A key reason why plant silicon and phytoliths research can take a leading role in forming the new Earth-life superdiscipline is that this phenomenon inherently and intimately links Earth and life. Silicon is the second most abundant element in the Earth's crust, whose uptake by plants affects biotic (Massey and Hartley, 2006; Epstein, 2009; Cooke and Leishman, 2011, 2016; Strömberg, 2011; Guntzer et al., 2012; Schoelynck et al., 2014; Hartley, 2015; Schoelynck and Struyf, 2016; Frew et al., 2016) and abiotic (Street-Perrott and Barker, 2008; Alexandre et al., 2011; Carey and Fulweiler, 2012, 2016; Song et al., 2012, 2017) processes at multiple scales. Understanding some of these processes requires and benefits from understanding the variation of plant silicon uptake and accumulation across taxa (Hodson et al., 2005; Katz, 2014, 2015; Strömberg et al., 2016), habitats, ecosystems and biomes (Carey and Fulweiler, 2012; Katz et al., 2013, 2014; Schoelynck et al., 2014; Song et al., 2017) and geologic time (Prasad et al., 2011; Strömberg, 2011; Katz, 2015; Strömberg et al., 2016). The references cited in this paragraph alone (and throughout this manuscript) demonstrate that many of us already carry out studies that cross and relax disciplinary boundaries, either in a single study or in a person's or group's combined research portfolio. It seems that this attribute of our field puts it in a better and more developed and advanced position to intimately merge Earth and life sciences, possibly even compared to some fields of research that lay deeper within the mainstream and that are more intensively studied, such as photosynthesis and the carbon cycle.

Hence, embedding superdisciplinary thinking in plant silicon and phytolith research can not only advance our field, but increase its impact in the merger of Earth and life sciences into a single superdiscipline. Working toward this goal is a true new frontier for plant silicon and phytolith research, for Earth-life sciences and for science in general.

## Author contributions

The author confirms being the sole contributor of this work and approved it for publication.

### Conflict of interest statement

The author declares that the research was conducted in the absence of any commercial or financial relationships that could be construed as a potential conflict of interest.
